# Fatigue of Narrow Dental Implants: Influence of the Hardening Method

**DOI:** 10.3390/ma13061429

**Published:** 2020-03-20

**Authors:** R.A. Pérez, J. Gargallo, P. Altuna, M. Herrero-Climent, F.J. Gil

**Affiliations:** 1 Bioengineering Institute of Technology, Universitat Internacional de Catalunya. C/ Josep Trueta s/n. Sant Cugat del Valles, 08195 Barcelona, Spain; rperezan@uic.es; 2 Faculty of Dentistry, Universitat Internacional de Catalunya, C/ Josep Trueta s/n. Sant Cugat del Valles, 08195 Barcelona, Spain; jgargallo@uic.es (J.G.); paltuna@uic.cat (P.A.); 3 Master Periodoncia, School of Dentistry, University of Seville, 41009 Seville, Spain; marianoherrero@herrerocliment.com

**Keywords:** narrow dental implants, mechanical properties, fatigue, titanium alloy, Ti–Zr alloy, hardening, fracture, hardness, commercially pure titanium, strain

## Abstract

The use of narrow titanium dental implants (NDI) for small ridges, reduced interdental space, or missing lateral incisors can be a viable option when compared to the conventional wider dental implants. Furthermore, in many cases, standard diameter implant placement may not be possible without grafting procedures, which increases the healing time, cost, and morbidity. The aim of this study was to analyze the mechanical viability of the current narrow implants and how narrow implants can be improved. Different commercially available implants (n = 150) were tested to determine maximum strength, strain to fracture, microhardness, residual stress, and fatigue obtaining the stress–number of cycles to fracture (SN) curve. Fractography was studied by scanning electron microscopy. The results showed that when the titanium was hardened by the addition of 15% of Zr or 12% cold worked, the fatigue limit was higher than the commercially pure grade 4 Ti without hardening treatment. Grade 4 titanium without hardening treatment in narrow dental implants can present fractures by fatigue. These narrow implants are subjected to high mechanical stresses and the mechanical properties of titanium do not meet the minimal requirements, which lead to frequent fractures. New hardening treatments allow for the mechanical limitations of conventional narrow implants to be overcome in dynamic conditions. These hardening treatments allow for the design of narrow dental implants with enhanced fatigue life and long-term behavior.

## 1. Introduction

Severe alveolar ridge reduction caused by periodontal diseases, trauma, or tooth loss can result in a reduced amount of bone in which to place regular diameter implants. In these cases, bone regenerative techniques have been highly described to increase the bone tissue volume simultaneously or before implant placement [1,2]. Despite this, reconstructive techniques are not exempt of limitations such as increasing healing time, cost, and patient morbidity [1].

Different studies indicate the need for a minimum bone space of 1.5 to 2 mm between a tooth and the implant and 3 mm between implants due to the tissues that should be accommodated, producing a correct function and esthetics [3].

The mechanical strength of commercially pure titanium is sometimes insufficient for the long lasting integrity of titanium implants [4], and consequently, the use of narrow dental implants represents a major risk of fractures [5,6,7]. Clinicians should be aware of the mechanical problems of dental implants, especially when the small diameters are used in zones subjected to high occlusal forces. Consequently, manufacturers have developed implants by increasing the strength in relation to the commercially pure Ti by two possibilities: (1) alloying the titanium with other biocompatible metals such as zirconium or niobium; and (2) straining the original commercially pure Ti by cold work.

It is well-known that the mechanical strength decreases very significantly with the diameter of the implant. A 3.5 mm diameter implant was 5.1 times weaker than a 5 mm diameter implant and 6.8 times weaker than a 6 mm diameter implant [4,8,9].

The design of dental implants always has to consider the cyclic loading during the service life of the implant, and therefore the fatigue endurance of the materials used play a very important role when trying to estimate the long term behavior of the device. The crack initiation by fatigue is on the surface of the dental implant. The crack growths into the dental implants were produced by the cyclic loads to fracture. The surface roughness produced by shot blasting and the compressive residual stress can favor the fatigue life of the dental implant. However, the connection surface where the load is applied and the strength of the material used are key parameters for the long-term mechanical properties of the dental implant [10,11,12]. Thus, the assessment of the fatigue behavior of implantable alloys has been taking greater importance. The materials used, fabrication process, and effective connection surface can be optimized in order to obtain a narrow dental implant with excellent static and dynamic mechanical properties. There is very little known about the fatigue of these narrow implants, and the durability and success rate have been described by only a limited number of clinical reports [10,13,14].

The objective of this study was to analyze the mechanical viability of current narrow implants and how narrow implants can be improved. This contribution presents a null hypothesis that the hardening treatments of the titanium do not have an influence on its mechanical behavior in the long-term.

## 2. Materials and Methods

### 2.1. Dental Implants.

**Figure 1:**
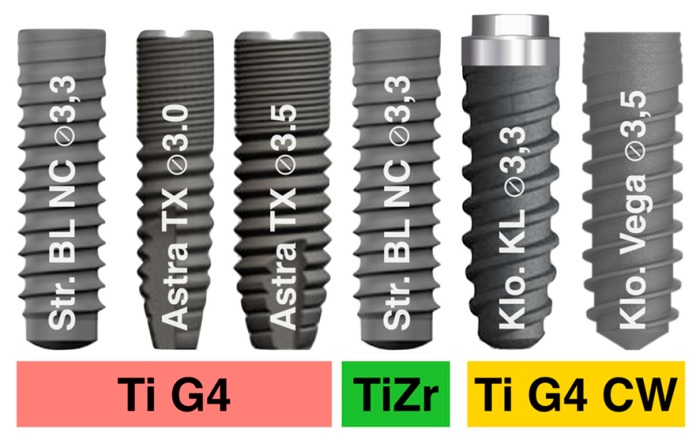
Narrow Dental Implants used in this in vitro study.

Different commercially available narrow dental implants were used (n = 150) in this study and distributed in three groups according to the type of titanium: Group 1, commercially pure grade 4 titanium; Group 2, titanium alloyed with 15% Zr; and Group 3, commercially pure grade 4 titanium hardened by 12% cold worked. The dental implants used are summarized in Table 1 and illustrated in Figure 1.

### 2.2. Mechanical Properties

Initially, static tension tests were conducted to determine the yield strength of the material, the ultimate strength, and the strain to fracture. The hardness of the specimens was measured using a Vickers microhardness tester (Akashi, Matsusawa, Japan) with a load of 100 gf and 15 s of indentation.

**Figure 2:**
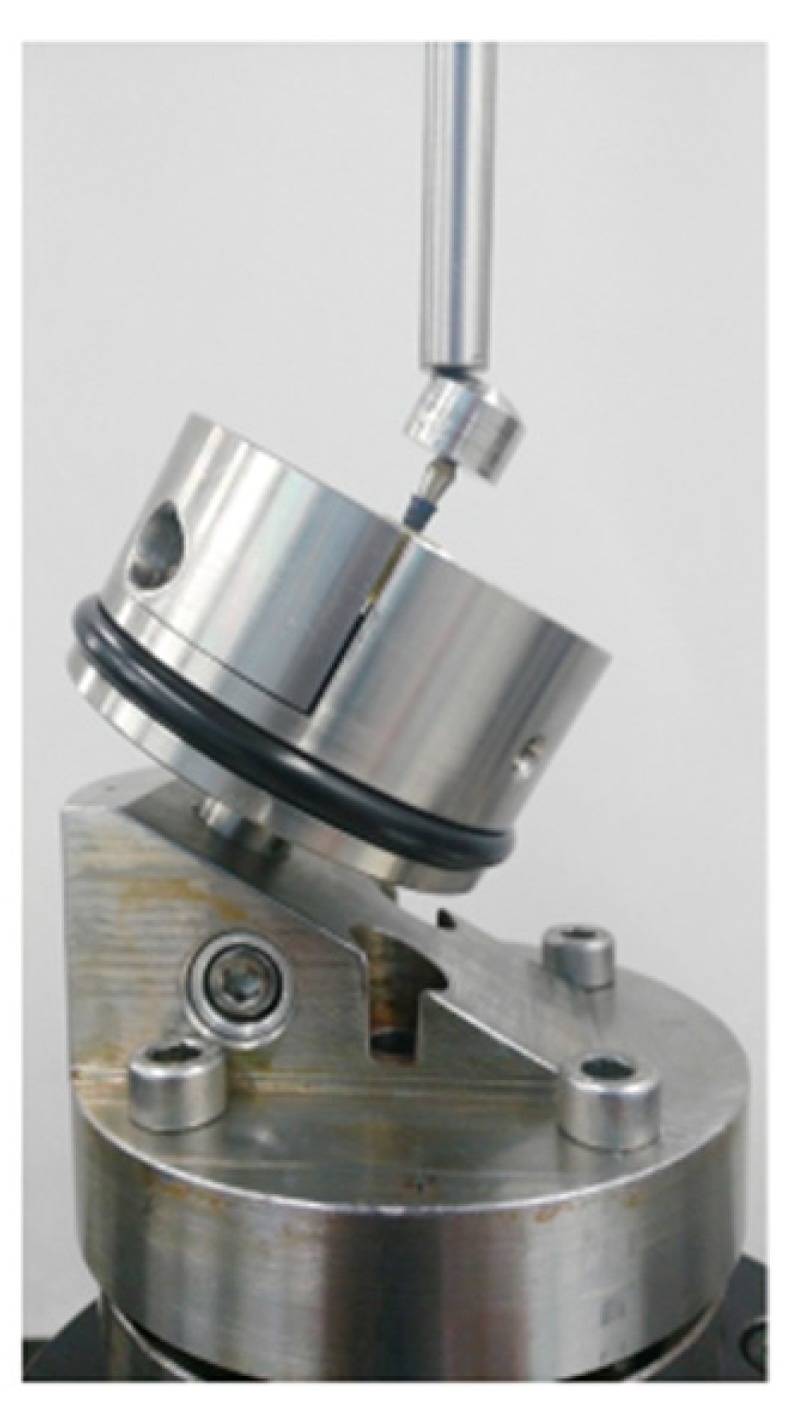
Clamp of the fatigue test machine.

Following the static tests, a fatigue test at various percentages of the obtained yield strength was performed, which allowed for the number of cycles before fracture to be determined. The aim was to find the stress value at which the sample supported a total of ten million cycles, which is considered to be the fatigue limit. The assays were performed with a servo-hydraulic testing machine (MTS Bionix 858, Minneapolis, MN, USA). This machine was equipped with a load cell MTS of 25 KN. The implants were loaded with a sinusoidal function of fatigue at a frequency of 15 Hz and 10% stress variation. The implants were fixed with an inclination of 30° with the axis z of the tensile-compression machine (Figure 2). The data are represented as the number of cycles reached at fracture for different applied stress. The deformed and fractured specimens were observed by means of scanning electron microcopy (JSM 6400, Jeol, Japan).

### 2.3. Residual Stress

Residual stresses were measured with a diffractometer incorporating a Bragg–Bentano configuration (D500, Siemens, Germany). The measurements were performed for the family of planes (213), which diffracted at 2θ = 139.5°. The elastic constants of Ti at the direction of this family of planes were EC = (E/1+ υ) _(213)_ = 90.3 GPa (1.4). The residual stress was designated as: σ = EC (1/d_0_) A; where d_0_ is the interplanar distance for the measuring angle Ψ = 0°.

### 2.4. Statistical Analysis

Statistically significant differences among the test groups for mechanical evaluation were assessed using statistical software (Minitab^TM^ 13.1, Minitab Inc., New York, USA). Analysis of Variance (ANOVA) tables with a multiple comparison Fisher test were calculated. The level of significance was established at a p-value < 0.005. Surgeons should be aware of the mechanical problems of dental implants, especially when small diameters are used in zones subjected to high occlusal forces

## 3. Results

The mechanical properties of the tested implants are shown in Table 2. The yield strength for the Grade 4 Ti did not differ among them, despite the difference in diameter. Nevertheless, their values became significantly higher when the implants were composed of Ti-15Zr or presented 12% cold work. A similar trend was observed for the maximum strength. On the other hand, the strain to fracture was lower for the Ti alloy and cold worked Ti. The Ti-15Zr presented a significantly higher value of strain to the cold worked Ti. The hardness was shown to be significantly higher for the cold worked Ti than the other conditions. Ti-15Zr also presented significantly higher hardness values than the Ti grade 4. Finally, the residual stresses were one order of magnitude higher for the cold work implants when compared to the other conditions.

The differences between the maximum strength of KL and Vega and the other implants were statistically significant (p < 0.005). Roxolid presented statistical differences with SLA, Yellow, and Aqua (p < 0.005). For the yield stress, the differences of the cold worked implants (KL and Vega) and the other implants were also statistically significant (p < 0.005). The same occurred with the hardness (p < 0.005) and the residual stress (p < 0.005) with the cold worked implants. However, the ductility was higher for SLA, Yellow, Aqua, and Roxolid, with differences statistically significant in relation to the cold worked dental implants.

**Figure 3:**
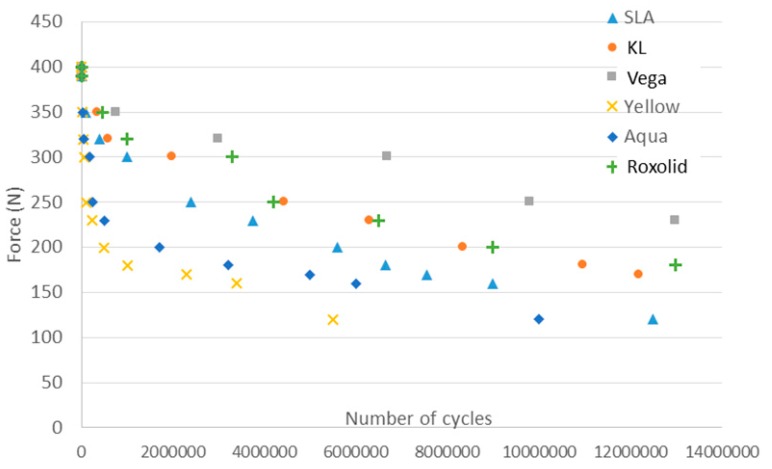
Stress–number of cycles to fracture (S–N) curve of the different narrow dental implants.

Figure 3 shows the S–N curve for the different dental implants. The dental implants are submitted at different forces (Y-axis) cycling from the compressive to unloaded. The number of the cycle when the dental implant is fractured is the value of the x-axis. The results show that the implants alloyed with zirconium and the grade 4 titanium submitted to 12% strained presented more fatigue life than the titanium dental implants (grade 4) without hardening treatment. Furthermore, as a general rule, it can be observed that bigger diameters tended to present a longer fatigue life.

**Figure 4:**
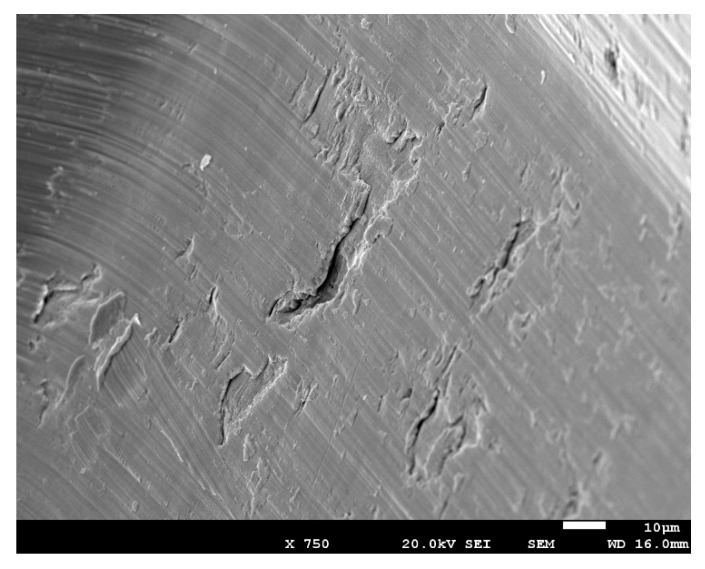
Crack nucleation in the connection place (dental implants with the abutment). In this zone, the mechanical load was the highest and the width of the dental implant was the lowest.

Fracture surfaces observed by means of scanning electron microscopy determined that the fracture was, in all cases, in the connection with the screw since it corresponded to the narrower part of the implant (Figure 4).

**Figure 5:**
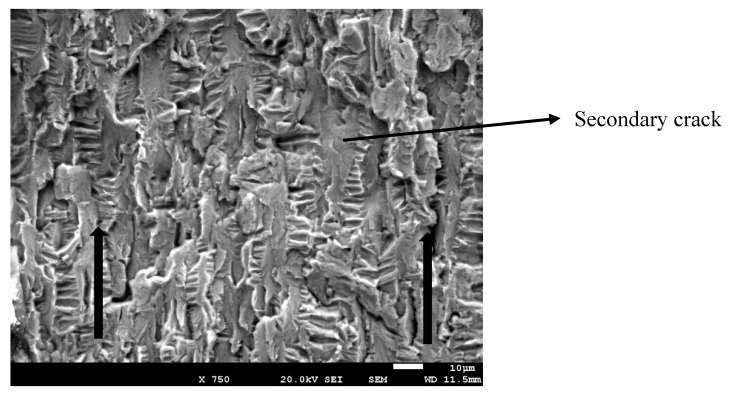
Crack propagation.

In Figure 5, the crack propagation can be observed that (arrows indicate the direction of the crack). The striated microstructure demonstrates the fatigue mechanism of the fracture. Furthermore, from Figure 5, secondary cracks can be observed, which were perpendicular to the direction of the propagation.

**Figure 6:**
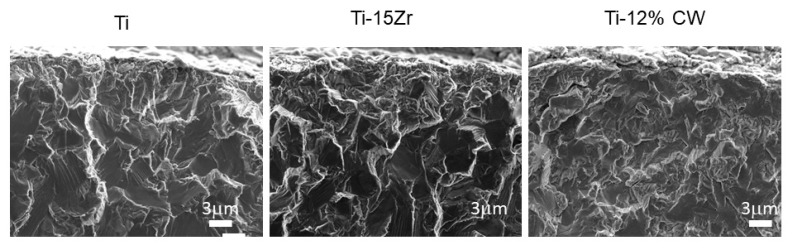
Fractography of the narrow dental implants.

The strained implants presented more percentage of the brittle fracture, and the other fractures were mainly ductile, corresponding to the grade 4 dental implants including the dental implants made with Ti15Zr (Figure 6). The narrow dental implants treated by cold work had lower ductility due to the difficulty of the dislocation movement. This produced lower damage of the fracture surface as can be observed in Figure 6. Ti-15Zr presented excellent mechanical properties (static and cyclic) without losing ductility.

## 4. Discussion

In clinical scenarios where insufficient bone or limited space is present, narrow sized implants may be required to replace the tooth lost. Narrow dental implants (3.3. to 3.5 mm) are well documented in all indications including load-bearing posterior regions. Smaller implants of 3.0 to 3.25 mm in diameter are well documented only for single-tooth non-load-bearing regions. Mini-implants <3.0 mm in diameter are only documented for the edentulous arch and single-tooth non-load-bearing regions, and success rates are not available [4,5,13,14]. Nevertheless, these implants may have severe medium- and long-term complications arising from their mechanical properties and lower resistance to fatigue [3,19]. Therefore, the mechanical properties of commonly used narrow implants, together with the masticatory process intrinsically bounded with continuous compressive loads, are important to consider when improving the current limitations of narrow dental implants, producing titanium alloys, or submitting Ti to cold work.

The grade 4 titanium narrow dental implants presented strength, yield stress, and hardness values lower than the strained or alloyed with 15% Zr dental implants, and only the ductility was higher than the treated implants.

Therefore, for high mechanical requirements, narrow dental implants cannot give a reliable response in their structural integrity.

The fatigue behavior of the narrow implants submitted to cold work was better due to the compressive effect of the residual stresses on the surface, which makes crack nucleation difficult. The cold work creates an important number of dislocations in the titanium, producing an increase in the hardness, compressive residual stress, and mechanical strength [20,21]. Similar values were obtained by the Ti-15Zr alloy due to the increase in mechanical strength [22,23,24]. The reason for this improvement was the distortion created in the crystalline structure by the substitution of titanium by zirconium atoms, producing difficulties for the movement of dislocations. These are the two mechanisms for increasing the mechanical properties of the narrow dental implants, as can be observed in the results in Table 2, where implants with the same diameter (Roxolid (TiZr alloy) and KL) present an important increase in the mechanical strength. It is also worth highlighting that KL present a hexagonal connection, which has been generally associated with low fatigue properties, compared to the internal conical connections [25].

Binon et al. [26] indicated tolerances of manufacturing as a reason for the described loose-fit of the prefabricated parts and requested the manufacturer to improve the fit of implant components. In loose-fit situations, the possibility of horizontal movement and micro rotation between the implant and abutment screw and lower the forces to tighten it, micromovements could lead to a progressive unscrewing of the abutment screw under dynamic loading conditions. The main cause of the high fatigue life of the external connection is due to the size of the resistant section; the external system presents a higher value of the area than the internal. This fact produces a better load distribution of the load and this is a main factor that explains the differences in the mechanical properties. The tolerances in the internal connections are better and favor the fatigue behavior of the internal connection system. However, this factor is not sufficient to improve the fatigue response in relation to the external connections. Raoofi et al. [15] used finite element analysis to ascertain that the stress concentration decreased when the internal surface area increased. In general, the place of fracture in the external connection is in the screw.

The results of Osseospeed Yellow presented the lowest limit of fatigue due to the narrowest dental implant diameter (3.0 mm) in comparison to the diameters of the Straumann (3.3 mm) and Aqua Astra-Tech (3.5 mm). The higher values of the Vega Klockner implant may be due to the increase in the diameter from 3.3 mm (Straumann-Roxolid) to 3.5 mm (Klockner-Vega). However, the mechanism of alloying produces an increase in the static mechanical properties as cold work, but with high values of strain to fracture, producing more toughness in the narrow dental implants [27,28].

Fracture was localized in all cases in the connection because it has a less effective diameter. Fractography revealed how crack propagation was very similar for the grade 4 and Ti-15Zr implants, where the crack propagation in the fracture surface presented ductility and the grooves of each cycle could be seen. The strained implants presented a fractography with brittle places. Consequently, cold work treatment produces an increase in the surface hardness, as shown in the results of the microhardness and residual compressive stress tests. This fact suggests that the crack nucleation site changes from the specimen’s surface (for the as-machined metal) to the specimen’s interior (for the strained metal). This behavior is also shown in grit blasting dental implants, which also improves the osseointegration [19,29,30,31]. This change is postulated to result in a significant modification of the fatigue properties of dental implants made of commercially pure Ti.

A limitation of this study is that the dental implants studied had slightly different designs and connections. We studied the commercial dental implants more widely used in the market within narrow dental implants. In the same way, we think that the most important factors in this study to determine fatigue life are the material and surface residual stresses of each dental implant.

This work will help clinicians make a more informed choice when choosing a small-diameter implant system. Narrow dental implants increase their fracture risk due to their smaller diameter, which might compromise the prosthetic components and also lead to bone overloading [31].

Abutment fracture is the primary prosthetic failure for two-piece narrow dental implants [30]. The narrower the implant diameter, the smaller the stress distribution area, which could contribute to the implant itself being more prone to damage accumulation [30]. It has been demonstrated that from narrow to standard and large diameter implants, an increasing probability of survival is observed with significant differences favoring cemented when compared to screw-retained prostheses [8,12].

## 5. Conclusions

Two methods for hardening the commercially pure titanium, cold working (12%) or alloying with 15% of zirconium, improved the mechanical properties, particularly the fatigue response of narrow implants. Implants with larger diameters showed higher limits of fatigue than narrower-implants. Commercially pure-Ti grade 4 should be studied by clinicians in order for the long-term success of the treatment. Narrow diameter implants, hardened with titanium–zirconium alloys or cold working, resulted in mechanical properties adequate for long-term behavior.

## Figures and Tables

**Table 1:** Implants used distributed by group [15,16,17,18].

Group	Group 1 Commercially Pure-Grade 4 Ti	Group 2 Ti Alloyed with 15% Zr	Group 3 Commercially Pure-Grade 4 Titanium Hardened by 12% Cold Worked
Implant Type	Bone level SLA (3.3 mm, h = 8 mm) (Straumann AGR, Basel, Switzerland) (n = 25) Bone level Osseospeed TX Yellow (3.0 mm, h =11 mm) (Astra Tech, Dentsply, Charlotte, NC, USA) (n = 25) Bone level Osseospeed TX Aqua (5 mm, h = 8 mm) (Astra Tech, Dentsply, Charlotte, North Carolina, US) (n = 25)	Bone level Roxolid (3.3 mm, h = 8 mm) (Straumann AGR, Basel, Switzerland) (n = 25)	KL (3.3 mm, h = 8 mm) with regular hexagon external connection (Klockner, Madrid. Spain) (n = 25). Bone Level Vega (3.5 mm, h = 8 mm) (Klockner, Madrid. Spain) (n = 25).
Connection Type	Conical internal	Cross-fit internal	Hexagon external/internal

**Table 2:** Mechanical properties of the dental implants studied include maximum strength and yield stress at 0.2%, expressed in megapascal (MPa), ductility is in percentages, the hardness is expressed in Vickers hardness number (HV) and residual stress in megapascal (MPa). The negative values of the residual stress represent the compressive nature of the stress. Standard deviation between parenthesis.

	Implant	Maximum Strength (MPa)	Yield Stress 0.2% (MPa)	Ductility (%)	Hardness (HV)	Residual Stress (MPa)
Group 1	SLA	520 (21)	443 (23)	16 (7)	109 (10)	−70 (6)
	Yellow	470 (40)	375 (12)	16 (5)	102 (10)	−54 (13)
	Aqua	480 (39)	368 (25)	19 (4)	103 (11)	−45 (12)
Group 2	Roxolid	887 (34)	689 (23)	24 (3)	197 (13)	−55 (17)
Group 3	KL	1032 (41)	783 (15)	6 (2)	356 (22)	−375 (43)
	Vega	1090 (37)	750 (21)	7 (3)	378 (26)	−398 (34)

## References

[1] Chiapasco M., Casentini P., Zaniboni M. (2009). Bone augmentation procedures in implant dentistry. Int. J. Oral Maxillofac. Implant..

[2] Chiapasco M., Casentini P., Zaniboni M., Corsi E., Anello T. (2012). Titanium-zirconium alloy narrow diameter implants for the rehabilitation of horizontally deficient edentulous ridges; prospective study on 18 consecutive patients. Clin. Oral Implant. Res..

[3] Grunder U., Gracis S., Capelli M. (2005). Influence of the 3-D bone-to-implant relationship on esthetics. Int. J. Periodontics Restor. Dent..

[4] Gargallo J., Satorres M., Puyuelo J.L., Sánchez-Garcés M.A., Pi-Urgell J., Gay-Escoda C. (2008). Endosseous dental implant fractures an analysis of 21 cases. Med. Oral Patol. Oral Cirugía Bucal.

[5] Goonarwardhana D., Judge R., Palamara J., Abduo J. (2016). Effect of implant diameter and alloy on peri-implant strain: An in vitro quantitative strain analysis. Eur. J. Prosthodont. Dent..

[6] Imam A., Moshaverinia A., McGlumphy A. (2014). Implant abutment interface: A comparison of the ultimate force to failure among narrow-diameter implant systems. J. Prosthet. Dent..

[7] Flanagan D. (2008). Fixed partial dentures and crows supported by small diameter dental implants in compromised sites. Implant Dent..

[8] Bordin D., Witek L., Fardin V.B., Bonfante E.A., Coelho P.G. (2018). Fatigue Failure of narrow implants with different implant-abutment connection designs. J. Prosthodont..

[9] Gil F.J., Planell J.A., Padrós A. (2002). Fracture and fatigue behaviour of shot blasted titanium dental implants. Implant. Dent..

[10] Gil F.J., Espinar E., Llamas J.M., Sevilla P. (2014). Fatigue life of bioactive titanium dental implants treated by means of Grit Blasting and Thermo-Chemical treatment. Clin. Implant. Dent. Relat. Res..

[11] Sevilla P., Sandino C., Arciniegas M., Martínez-Gomis J., Peraire M., Gil F.J. (2010). Evaluating mechanical properties and degradation of YTZP dental implants. Mater. Sci. Eng. C.

[12] Gil F.J., Herrero-Climent M., Lázaro P., Rios J.V. (2014). Implant–abutment connections: Influence of the design on the microgap and their fatigue and fracture behavior of dental implants. J. Mater. Sci. Mater. Med..

[13] Karl M., Krafft T., Kelly J.R. (2014). Fracture of a narrow-diameter roxolid implant: Clinical and fractographic considerations. Int. J. Oral Maxillofac. Implant..

[14] Tolentino L., Sukekava F., Seabra M., Lima L.A., Garcez-Filho J., Araujo M.G. (2014). Success and survival rates of narrow diameter implants made of titanium-zirconium alloy in the posterior region o. the jaws-results from a 1 year follow-up. Clin. Oral Implant. Res..

[15] Raoofi S., Khademi M., Amid R., Kadkhodazadeh M., Moyahhedi M.R. (2013). Comparison of the effect of three abutment-implant connections on stress distribution the internal surface on dental implants: A Finite element Analyis. J. Dent. Res. Clin. Dent. Prospect..

[16] Alemida E.O., Freitas Ac Bonfante E.A. (2013). Mechanical testing of implant supported anterior crowns with different implant/abutment connections. Int. J. Oral Maxillofac. Implant..

[17] Shim H.W., Yang B.E. (2015). Long-term cumulative survival and mechanical complications of single tooth Ankylos Implants: Focus on the abutment neck fractures. J. Adv. Prosthodont..

[18] Godoy-Gallardo Wang Z., Shen Y., Manero J.M., Gil F.J., Rodriguez D., Haapasalo M. (2015). Antibacterial Coatings on Titanium Surfaces: A Comparison Study Between in Vitro Single-Species and Multispecies Biofilm. ACS Appl. Mater. Interfaces.

[19] Aparicio C., Rodriguez D., Gil F.J. (2011). Variation of roughness and adhesion strength of deposited apatite layers on titanium dental implants. Mater. Sci. Eng. C.

[20] Gil F.J., Planel J.A., Padros A., Aparicio C. (2007). The effect of shot blasting and heat treatment on the fatigue behavior of titanium for dental implant applications. Dent. Mater..

[21] Altuna P., Lucas- Taulé E., Gargallo-Albiol J., Figueras-Alvarez O., Hernández-Alfaro F., Nart J. (2016). Clinical evidence on titanium-zirconium dental implants: A systematic review and meta-analysis. Int. J. Oral Maxillofac. Surg..

[22] Dohan D., Vazquez L., Park Y., Sammartino G., Bernard J.P. (2011). Identification card and codification of the chemical and morphological characteristics of 14 dental implant surfaces. J. Oral Implant..

[23] Nelson K., Schmelzeisen R., Taylor T.D., Zabler S., Wiest W., Fretwurst T. (2016). The impact of force transmission on narrow-body dental implants made of commercially pure titanium and titanium zirconia alloy with a conical implant-abutment connection: An experimental pilot study. Int. Oral Maxillofac. Implant..

[24] Takahashi M., Kikuchi M., Takada Y. (2016). Mechanical properties and microstructures of dental cast Ti-6Nb-4Cu, Ti-18Nb-2Cu, and Ti-24Nb-1Cu alloys. Dent. Mater. J..

[25] Hirata R., Bonfante E., Machado L., Tovar N., Coelho P.G. (2014). Mechanical evaluation of four narrow-diameter implant systems. Int. J. Prosthodont..

[26] Binon P.P., Curtis D.A. (2007). A classification system to measure the implant-abutment microgap. Int. J. Oral Maxillofac. Implant..

[27] Pegueroles M., Tonda-Turo C., Planell J.A., Gil F.J., Aparicio C. (2012). Adsorption of fibronectin, fibrinongen, and albumin on TiO2: Time-Resolved Kinetics, Structural Changes, and Competition Study. Biointerphases.

[28] Manero J.M., Gil F.J., Padros E., Planell J.A. (2003). Applications of environmental scanning electron microscopy (ESEM) in biomaterials field. Microsc. Res. Tech..

[29] Allum S.R., Tomlinson R.A., Joshi R. (2008). The impact of loads on standard diameter, small diameter and mini implants: A comparative laboratory study. Clin. Oral Implant. Res..

[30] Nicolas-Silvente A.I., Velasco-Ortega E., Ortiz-García I., Monsalve-Guil L., Gil F.J., Jimenez-Guerra A. (2020). Influence of the Titanium implants surface treatment on the surface roughness and chemical composition. Materials.

[31] Velasco-Ortega E., Flichy-Fernández A., Punset M., Jiménez-Guerra A., Manero J.M., Gil F.J. (2019). Fracture and Fatigue of Titanium Narrow Dental Implants: New Trends in Order to Improve the Mechanical Response. Materials (Basel).

